# Intracerebral Hemorrhage after Intravenous Thrombolysis in Patients with Cerebral Microbleeds and Cardiac Myxoma

**DOI:** 10.3389/fneur.2014.00252

**Published:** 2014-12-01

**Authors:** Aurauma Chutinet, Duangnapa Roongpiboonsopit, Nijasri C. Suwanwela

**Affiliations:** ^1^Faculty of Medicine, Department of Medicine, Division of Neurology, Chulalongkorn University, King Chulalongkorn Memorial Hospital, Thai Red Cross Society, Bangkok, Thailand; ^2^Faculty of Medicine, Department of Medicine, Naresuan University, Phitsanulok, Thailand

**Keywords:** microbleeds, cardiac myxoma, thrombolysis, recanalization

## Abstract

**Background and purpose:** Cardiac myxoma is a rare etiology of stroke. Both cerebral microbleeds and cardiac myxoma may increase the risk of intracerebral hemorrhage after intravenous (IV) thrombolysis. However, data are still limited. We report a case of multiple cerebral microbleeds treated with IV thrombolysis with later findings of cardiac myxoma.

**Summary of case:** A 58-year-old-man presented with right-sided hemiplegia and global aphasia. The presumptive diagnosis of acute left middle cerebral artery (MCA) infarction was made. Previous magnetic resonance imaging showed multiple cerebral microbleeds. The patient received IV thrombolysis. Bilateral cerebellar hemorrhage occurred after thrombolysis, and a median suboccipital craniectomy and hematoma removal was performed. Transthoracic echocardiogram found a left atrial myxoma. The tumor was then surgically removed. Six months later, neurological deficit improved.

**Conclusion:** Cerebral microbleeds may be associated with atrial myxoma. IV thrombolysis could benefit acute ischemic stroke patients with both baseline cerebral microbleeds and atrial myxoma.

## Introduction

Cardiac myxoma is a rare etiology of stroke. Both cerebral microbleeds and cardiac myxoma may increase the risk of intracerebral hemorrhage (ICH) after IV thrombolysis (1–4). However, data are still limited. We report a patient with multiple cerebral microbleeds who was treated with thrombolysis and was later found to have cardiac myxoma.

## Case Review

**Figure 1:**
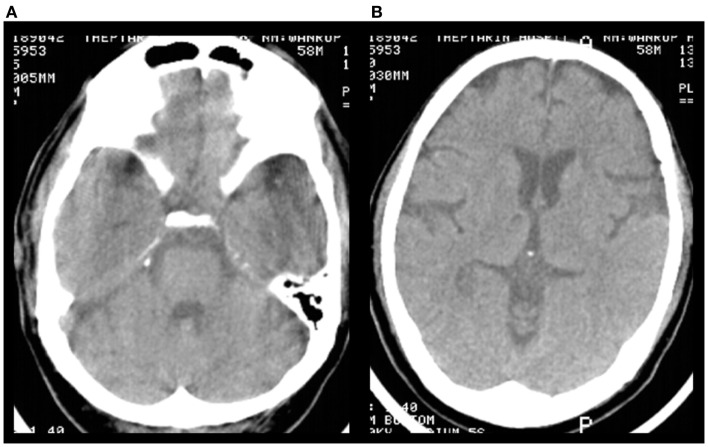
**Pre-treatment non-contrast brain CT scan**. **(A,B)** There was no evidence of acute ischemic or hemorrhagic stroke.

**Figure 2:**
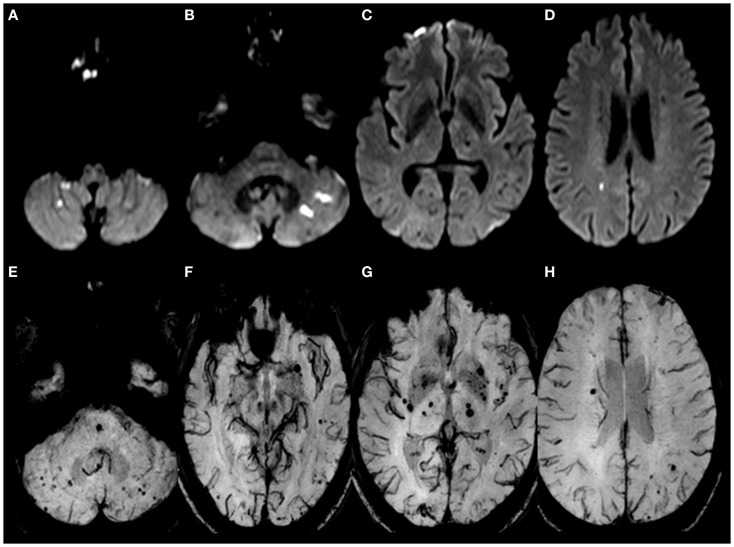
**Brain MRI performed during the previous ischemic stroke 4 months earlier**. **(A–D)** DWI showed restricted fluid diffusion in cerebellar hemispheres, left temporal cortex, right parietal deep white matter consistent with acute infarctions. **(E–H)** SWI-MRI (susceptibility weighted imaging MRI) showed multiple cerebral microbleeds, particularly in the deep gray matter and posterior fossa, possibly due to hypertensive vasculopathy.

**Figure 3:**
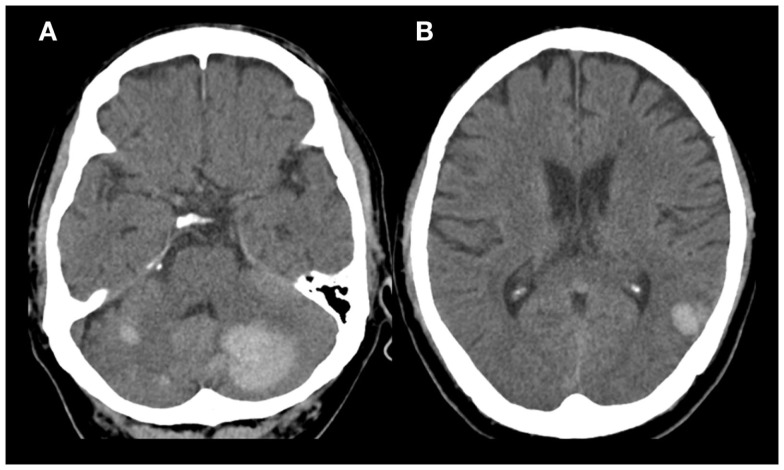
**Non-contrast brain CT scan 1 day after thrombolysis**. **(A)** Round shaped (3.1 cm × 3.2 cm × 2.3 cm) hyperdense lesion occupying the left cerebellum and foci of hyperdense lesion in right cerebellum. **(B)** Foci of hyperdense lesion in left temporal lobe.

**Figure 4:**
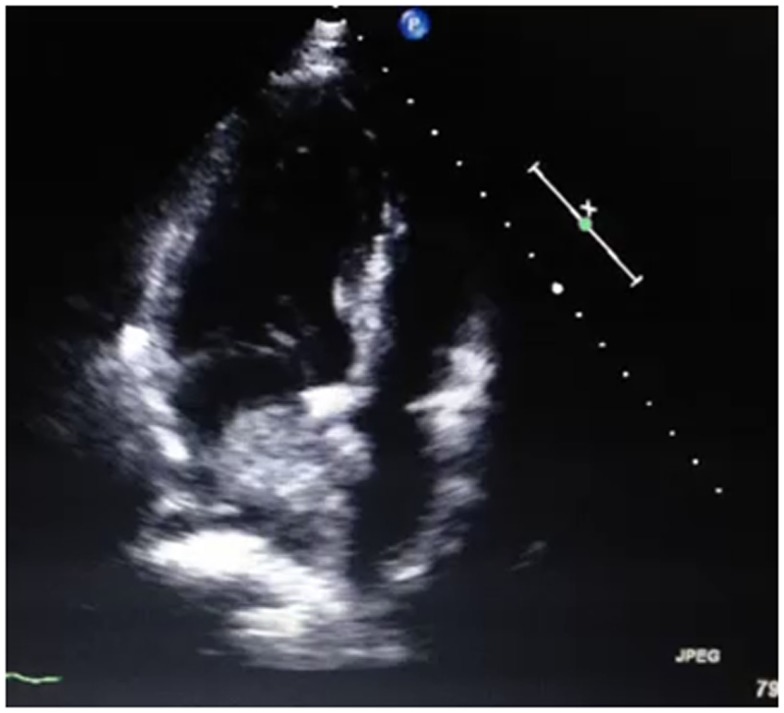
**Transthoracic echocardiogram findings**. A large (55 mm × 25 mm) mobile and lobulated heterogeneous echogenic mass with scatter calcification attached to interatrial septum and in and out protrusion of mitral annular plane, highly mobile with thrombus on top. Findings were consistent with left atrial myxoma with superimposed thrombus.

**Figure 5:**
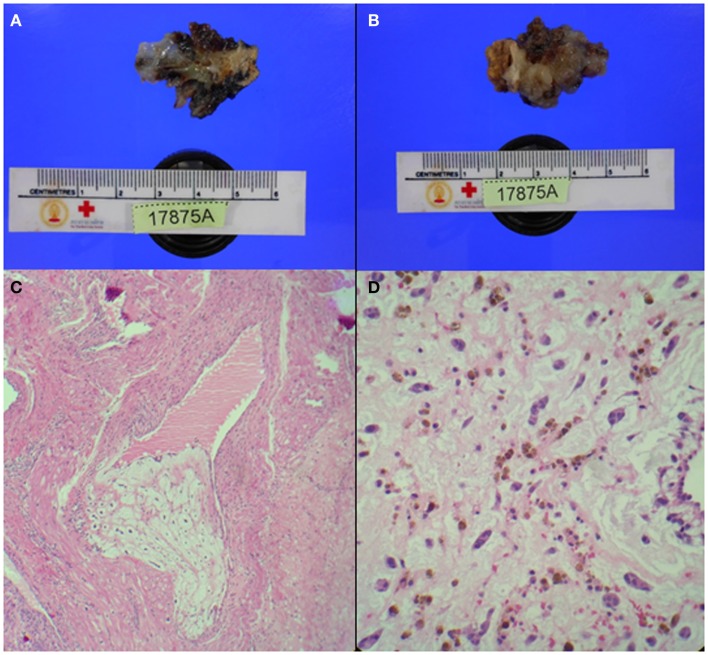
**Pathology findings**. **(A,B)** Gelatinous tissue (4 cm × 2.5 cm × 0.8 cm) attached to part of tumor stalk (1.5 cm × 1 cm × 0.5 cm). **(C)** Extension of tumor tissue into trabecule and venous channel. **(D)** Tumor cells: spindle or stellate shape, eosinophilic cytoplasm, and bland-looking nuclei. Tumor cells were located in myxoid stroma and surrounded by numerous red blood cells.

A 58-year-old Thai male with hypertension and previous ischemic stroke 4 months earlier, developed a sudden onset of right-sided weakness and was unable to speak 2 h prior to hospital arrival. His blood pressure was well controlled (<140/90 mmHg) with amlodipine 5 mg/day before and after his previous ischemic stroke. Aspirin 81 mg and simvastatin 20 mg/day were given after previous stroke. At the emergency room, his blood pressure was 158/89 mmHg and pulse was 82 beats per minute (regular) with regular heart rate and rhythm. There were no cardiac murmurs on auscultation. The patient was alert but had global aphasia with forced eye deviation to the left. Right facial weakness (upper motor neurons), right hemiplegia, right hypoanesthesia, and right homonymous hemianopia were found. The NIH Stroke Scale (NIHSS) was 20. Complete blood cell count, coagulation profile, plasma level of glucose, electrolytes, renal and liver function tests were normal. An electrocardiogram demonstrated normal sinus rhythm with a rate of 80 beats per minute. Non-contrast computed tomography (CT) scan of the head demonstrated no evidence of acute ischemic or hemorrhagic stroke (Figure 1). The diagnosis of acute left MCA infarction was made and cardiogenic embolism was highly suspected. Patient’s magnetic resonance imaging (MRI) and magnetic resonance angiography (MRA) performed after the previous stoke 4 months earlier were reviewed and demonstrated infarction in both cerebellar hemispheres and right parietal deep white matter area. Multiple microbleeds were also noted predominantly at basal ganglia, thalamus, and cerebellum, bilaterally (Figure 2). Despite the microbleeds on the previous MRI, the stroke neurologist felt that there was no absolute contraindication for intravenous (IV) thrombolysis. Therefore, IV tissue plasminogen activator was given 140 min after stroke onset. Computed tomography angiography (CTA) and perfusion study (CTP) were subsequently performed 1 h after thrombolysis treatment initiation. CTP showed a delayed time to peak (TTP) and mean transit time (MTT) with preservation of cerebral blood volume (CBV) at the left parieto-occipital region, which is likely to indicate the presence of ischemic penumbra. However, no significant stenosis of MCA was found on CTA thus mechanical thrombectomy was not performed. Standard post-IV thrombolysis care protocol, including close blood pressure monitoring, was carried out. Patient continued to have right hemiplegia and global aphasia with a NIHSS of 20. A non-contrast CT scan of the brain 24 h after thrombolysis revealed a hematoma at the left cerebellum with perilesional edema and multiple foci of hyperdense lesion at right cerebellum, superior frontal gyrus, and left temporal lobe consistent with hemorrhagic transformation (parenchymal hematoma type 2) (5) (Figure 3). Patient symptoms worsened between 24 and 48 h post-IV thrombolysis, he was stuporous and NIHSS progressed from 20 to 22. Repeated CT brain 3 days after thrombolytic treatment showed worsening of the left cerebellar edema. Patient underwent a median suboccipital craniectomy and hematoma evacuation and thereafter his condition resulted as clinically stable without evidence of brainstem compression. As part of the protocol for the etiologic diagnosis of stroke, a transthoracic echocardiogram (TTE) was performed revealing a large (55 mm × 25 mm) mobile, lobulated, heterogeneous echoic mass with scattered calcification attached to interatrial septum with in and out protrusion of the mitral annular plane. A highly mobile component at the surface of left atrial mass was also present. Findings were consistent with both left atrial myxoma and superimposed thrombus (Figure 4). After discussion of the clinical case, informed consent was given by the patient’s family to surgically remove the atrial myxoma. Pathological examination confirmed the diagnosis of myxoma. There was a piece of gelatinous tissue (4 cm × 2.5 cm × 0.8 cm) attached to a part of tumor stalk. Histopathological examination revealed that the tumor extended into the venous channel on underlying myocardium. The resection margin was free of tumor (Figure 5) and after 31 days of admission, the patient was discharged to return home. At time of discharge, he still had right hemiplegia and global aphasia, NIHSS was 16, Barthel index 0, and the modified Rankin Scale (mRS) 5. After 6 months, his clinical condition was significantly improved, including gait and speech. NIHSS was 7, Barthel index 25, and mRS 4. At 9 months, the patient was able to engage in walking without assistance, the NIHSS was 5, Barthel index 65, and mRS 3.

## Discussion

We report a case of IV thrombolysis treatment in patient with atrial myxoma and cerebral microbleeds. The patient had a previous ischemic stroke 4 months earlier and was known to have multiple cerebral microbleeds in bilateral deep gray matter and posterior fossa on MRI. He developed acute ischemic stroke in the MCA territory and received IV thrombolysis despite the presence of multiple cerebral microbleeds. Investigations later found that he had atrial myxoma, which may have been the cause of the microbleeds. Another possible cause of cerebral microbleeds is his history of hypertension. However, the patient had good control of his blood pressure. He developed symptomatic intracerebral hemorrhage (sICH) after thrombolytic treatment (6), which required surgical decompression.

This case has three major points of interest. Firstly, it supports the previous finding that cerebral microbleeds and previous stroke increase the risk of ICH after thrombolysis. Our patient developed sICH 1 day after receiving IV thrombolysis, which is consistent with results from two meta-analyses showing trends of increased risk of sICH after thrombolysis in patients with microbleeds (3, 4). Moreover, there was a significant association between increased cerebral microbleeds burden and the risk of any ICH and sICH after thrombolysis (1). Our patient also had high burden of cerebral microbleeds (>10 microbleeds).

For bleeding location after thrombolysis, a small study of eight acute ischemic stroke patients with cerebral microbleeds receiving thrombolysis found that the hemorrhage always occurs at the area of the ischemic stroke, whereas none of the ICH occurred at the site of cerebral microbleeds (7). In our patient, ICH was found in the cerebellum at the site of cerebral microbleeds and at the site of previous ischemic area. Although the cerebral microbleeds in this patient may be attributable to hypertension, the site of microbleeds is unusual. Microbleeds in the cerebellum are found in 17% of cases; moreover, microbleeds in the cerebellum are likely to be located medially near dentate nuclei (8). In our patient, microbleeds were found to be located peripherally in the cerebellar hemisphere.

To date, the risk of ICH after thrombolysis in patients with cerebral microbleeds is still uncertain and there are no recommendations to prevent patients from receiving IV thrombolysis in the presence of cerebral microbleeds on MRI (9). Although there is no definite contraindication for thrombolysis in our patient according to the current guidelines, special precaution is needed in patients with history of previous stroke and microbleeds especially if the cause of microbleeds is related to atrial myxoma.

The second and probably more important aspect of this clinical case is the cause of stroke and microbleeds. Cerebral microbleeds has been detected in several medical conditions such as hypertensive vasculopathy, cerebral amyloid angiopathy (CAA), diffuse axonal injury after head trauma, or cardiac myxoma (10, 11). Clinical course of patients and distribution of cerebral microbleeds can be helpful for differentiating the etiology of cerebral microbleeds. Cerebral microbleeds in the basal ganglia, thalamus, brainstem, and cerebellum typically result from hypertensive vasculopathy. However, our patient had his hypertension well under control and also had cerebral microbleeds located in the peripheral areas of the brain. CAA, which mostly occurs in the elderly and where multiple cerebral microbleeds are usually restricted to lobar, cortical, or cortico-subcortical areas, especially posterior cortical regions, is unlikely in our patient. This patient did not have previous head injury, and microbleeds were not located in the frontal and temporal regions, hence we can rule out head trauma-related diffuse axonal injury.

Atrial myxoma is a rare cause of stroke. Cerebral embolism leading to ischemic stroke, aneurysmal formation, and myxomatous metastasis are common neurological complications. In this case report, we found that multiple microbleeds and recurrent territorial infarction can be associated with the presence of as atrial myxoma. To our knowledge, there is only one report of microbleeds as a complication of atrial myxoma (12). Pathological findings of the brain in patients with atrial myxoma are generally consistent with multiple infarctions. Aneurysmal formation and pseudoaneurysms can be found at the site of vascular occlusion. It has been proposed that tumor cells may infiltrate cerebral vessels via vasa vasorum and subsequently proliferate in the vessel wall, leading to a weakening of the internal elastic lamina and aneurysmal formation. This process is similar to the mechanism underlying the formation of mycotic aneurysms. Sometimes, rupturing of the three layers of the arterial wall occurs and pseudoanerysms are formed. These pseudoaneurysms generally grow faster than the true aneurysm itself and can result in hemorrhage. Microembolism has also been demonstrated in the retina of patients with atrial myxoma (13). We proposed that small myxoma particles, which embolize and lodge in the small vessels in the brain may lead to formation of microbleeds.

The third and possibly the most important point in this patient is the risk of bleeding after thrombolysis in patients with myxoma and cerebral microbleeds. Embolized myxoma particles generally lodge in brain arteries and proliferate in the vessel wall. Pseudoaneurysm and aneurysm formation in the small arteries may be more vulnerable to thrombolysis induced ICH. There were nine reports of patients, including ours, with acute ischemic stroke due to cardiac myxoma who received IV thrombolysis (3, 4, 14–19) (Table 1). All patients developed ischemic stroke in the MCA territory and received IV thrombolysis within 3 h after stroke onset. Three out of nine patients, including our patient, developed ICH after IV thrombolysis. Bleeding was found located in the cerebellum and parietal lobe. Our patient was the only case with reported evidence of multiple cerebral microbleeds on MRI. Other authors did not report any data on microbleeds. Among these case reports, most patients, including ours, improved after thrombolysis and had a good clinical outcome. Due to the invasive nature of embolized myxomatous material in the cerebral vessel, special precaution should be taken with IV thrombolysis in patients with a diagnosis of atrial myxoma, especially in those with evidence of cerebral microbleeds.

**Table 1:** **Characteristics of patients with acute ischemic stroke due to cardiac myxoma receiving intravenous thrombolysis**.

Reference	Year	Age/sex	OTT min	NIHSS	Location	ICH within 7 days of stroke onset	Outcome
Chong (3)	2005	74/F	180	6	Left MCA	Yes	Aphasia
						Cerebellum	
						Left parietal SAH	
Ibrahim (14)	2008	51/M	84	22	Left MCA	No	Complete recovery
Nagy (15)	2009	26/M	105	10	Right MCA	No	Minimal hand weakness at 2 years
Ong (16)	2011	22/F	125	12	Right MCA	No	NIHSS 5 at 10 months
Sun (17)	2011	45/M	172	16	Left MCA	No	Motor aphasia
Acampa (4)	2011	63/F	160	19	Right MCA	Yes	Slight improved NIHSS 15 at day 25
						Right parietal	
Ruzicka – Kaloci (18)	2012	42/F	−	17	Left MCA	No	NIHSS 8
Hatayama (19)	2012	76/M	155	−	Left MCA	No	Improved
Our case report	2014	58/M	140	20	Left MCA	Yes	Improved at 6 months
						Cerebellum	Able to walk with assistance
							Some aphasia

*ICH, intracerebral hemorrhage; MCA, middle cerebral artery; NIHSS, National Institute of Health Stroke Scale; OTT, onset to treatment time; SAH, subarachnoid hemorrhage*.

We propose that in case of involvement of several arterial territories, physicians should consider cardioembolic stroke in the differential diagnosis and continue aggressive work up, to adopt the most appropriate therapeutic strategies and prevent recurrent stroke. Ischemic stroke patients with cerebral microbleeds and atrial myxoma may have an increased risk of bleeding from IV thrombolysis.

## Conflict of Interest Statement

The authors declare that the research was conducted in the absence of any commercial or financial relationships that could be construed as a potential conflict of interest.
